# Supplementary material for: Metabolomic and Mitochondrial Fingerprinting of the Epithelial-to-Mesenchymal Transition (EMT) in Non-Tumorigenic and Tumorigenic Human Breast Cells
Source: Cancers (Basel). 2022 Dec 16;14(24):6214. doi: 10.3390/cancers14246214 (PMC9776482; doi:10.3390/cancers14246214)
Supplement: Supplementary file 1 [file cancers-14-06214-s001.zip › cancers-2047273-supplementary.pdf]

Metabolomic and Mitochondrial Fingerprinting of the Epithelial-to-Mesenchymal Transition (EMT) in Non-Tumorigenic and Tumorigenic Human Breast Cells

SUPPLEMENTAL INFORMATION

Table S1. Concentration of bioenergetic metabolites in HMLE/HMLE-EMT and HMLER/HMLER-EMT cells

Metabolite	HMLE	HMLE-EMT	HMLER	HMLER-EMT
Pyruvate[c,d]	3.8 (0.6)	2.4 (0.6)	2.7 (0.6)	21.4 (2.0)
Lactate[f]	78 (9)	83 (5)	86 (4)	551 (57)
Alanine[c,f]	48 (12)	102 (12)	45 (6)	122 (22)
Valine[a,e]	328 (17)	574 (43)	304 (17)	361 (43)
Leucine[a,d]	428 (39)	676 (19)	493 (41)	419 (27)
Isoleucine[a,e]	202 (28)	366 (11)	211 (28)	280 (30)
Succinate[c]	6.4 (0.6)	5.9 (1.4)	7.1 (0.8)	3.7 (0.7)
Fumarate[c,f]	0.7 (0.2)	2.3 (0.4)	0.9 (0.1)	5.4 (0.5)
Serine[a,e]	516 (30)	959 (174)	502 (41)	632 (65)
Oxaloacetate[f]	5.5 (1.3)	9.6 (0.6)	9.6 (1.9)	21.6 (2.7)
Malate[a,e,f]	1.6 (0.1)	3.5 (0.9)	1.9 (0.4)	13.9 (0.8)
Aspartate[a,c,f]	124 (8)	182 (30)	134 (12)	215 (24)
Phosphoenolpyruvate[a,c,f]	0.78 (0.25)	10.40 (0.63)	1.96 (0.24)	84.67 (4.02)
Glutamate[a,f]	183 (16)	332 (29)	204 (25)	313 (21)
Glutamine	184 (167)	3490 (2215)	621 (321)	1512 (2742)
Glyceraldehide 3-P	ULOD	ULOD	ULOD	49 (8)
3-phosphoglycerate[a,c,f]	14.6 (0.8)	62.2 (10.2)	24.2 (5.8)	209.0 (7.7)
Citrate[d]	17.0 (3.4)	25.7 (4.0)	21.1 (2.4)	7.5 (0.6)
Glucose[a,f]	6.4 (1.5)	22.7 (3.7)	8.5 (1.5)	24.2 (2.8)
Ribose 5-P[d]	5.7 (1.4)	2.9 (0.5)	6.8 (0.6)	11.0 (1.9)
Fructose 6-P[a,e,f]	2.7 (0.8)	34.5 (2.4)	6.2 (1.3)	12.6 (1.8)
Glucose 6-P[a]	1.4 (0.1)	42.3 (3.0)	5.6 (1.2)	5.2 (0.3)
Fructose 1,6-BisP[a,b,d]	2.1 (0.4)	34.9 (2.2)	5.8 (1.2)	4.5 (0.9)
6-P-gluconate[a,d]	20.6 (2.5)	33.0 (4.1)	24.3 (1.3)	21.9 (1.3)
3-hydroxybutyrate[a,e,f]	55 (9)	136 (7)	70 (6)	102 (15)
Succinyl-CoA[c]	10.4 (1.8)	10.5 (3.1)	8.0 (1.1)	16.2 (1.9)
Malonyl-CoA[a,e,f]	12.6 (2.1)	32.2 (4.5)	15.8 (2.3)	24.7 (2.9)
α-ketoglutarate	ULOD	ULOD	ULOD	0.25 (0.03)
2-hydroxyglutarate	ULOD	ULOD	ULOD	21 (2)

Concentrations are shown in μM/mg of protein $\pm$ SEM (n=5, in triplicate)
Decimals were set according to the first significant digit of the measured SEM

[a] Changes between HMLE and HMLE-EMT are statistically significant (p-value < 0.05)
[b] Changes between HMLE and HMLER are statistically significant (p-value < 0.05)
[c] Changes between HMLER and HMLER-EMT are statistically significant (p-value < 0.05)
[d] Changes between HMLE-EMT and HMLER-EMT are statistically significant (p-value < 0.05)
[e] Changes between HMLER and HMLE-EMT are statistically significant (p-value < 0.05)
[f] Changes between HMLE and HMLER-EMT are statistically significant (p-value < 0.05)

ULOD: Upper Limit of Detection

Table S2. Concentration of homocysteine-methionine 1C metabolites in HMLE/HMLE-EMT and HMLER/HMLER-EMT cells

Metabolite	HMLE	HMLE-EMT	HMLER	HMLER-EMT
Cystathionine[a,c,d]	0.0034 (0.0008)	0.0012 (0.0004)	0.0021 (0.0005)	0.0087 (0.0013)
Cysteine[d]	4.2 (0.6)	1.9 (0.4)	ULOQ	9.6 (1.3)
Betaine[a,e]	0.044 (0.004)	0.028 (0.001)	0.042 (0.008)	0.041 (0.008)
Choline	2.4 (0.5)	2.3 (0.3)	2.3 (0.3)	2.3 (0.4)
Dimethylglycine	2.3 (0.2)	2.3 (0.3)	2.2 (0.3)	2.4 (0.5)
Homocysteine[a]	0.19 (0.03)	0.72 (0.06)	0.29 (0.01)	0.28 (0.07)
Met[a,e,f]	0.61 (0.10)	2.10 (0.36)	0.89 (0.10)	1.24 (0.20)
SAM[a,c,d]	0.031 (0.006)	0.008 (0.002)	0.014 (0.002)	0.099 (0.023)
AMP	0.036 (0.012)	0.031 (0.002)	0.034 (0.007)	0.034 (0.005)
NADH[a,c,d]	0.46 (0.03)	0.32 (0.04)	0.40 (0.03)	2.82 (0.56)
SAH[b,c,d]	0.014 (0.004)	0.006 (0.002)	0.004 (0.001)	0.039 (0.004)
Riboflavin[a,e]	0.015 (0.003)	0.033 (0.006)	0.017 (0.001)	0.020 (0.002)
Gly[a]	98 (15)	217 (9)	138 (6)	143 (7)
Ser[a,d]	501 (67)	943 (98)	537 (53)	528 (42)

Concentrations are shown in µM/mg of protein ± SEM (n=5, in triplicate)
Decimals were set according to the first significant digit of the measured SEM

[a] Changes between HMLE and HMLE-EMT are statistically significant (p-value < 0.05)
[b] Changes between HMLE and HMLER are statistically significant (p-value < 0.05)
[c] Changes between HMLER and HMLER-EMT are statistically significant (p-value < 0.05)
[d] Changes between HMLE-EMT and HMLER-EMT are statistically significant (p-value < 0.05)
[e] Changes between HMLER and HMLE-EMT are statistically significant (p-value < 0.05)
[f] Changes between HMLE and HMLER-EMT are statistically significant (p-value < 0.05)

Met: methione; SAM:5-adenosyl-methionine; AMP:3' 5' adenosine monophosphate; NADH; nicotinamide adenine dinucleotide phosphate, reduced; SAH: 5-adenosyl-homocysteine; Gly: glycine; Ser: serine; ULOD: Upper Limit of Detection

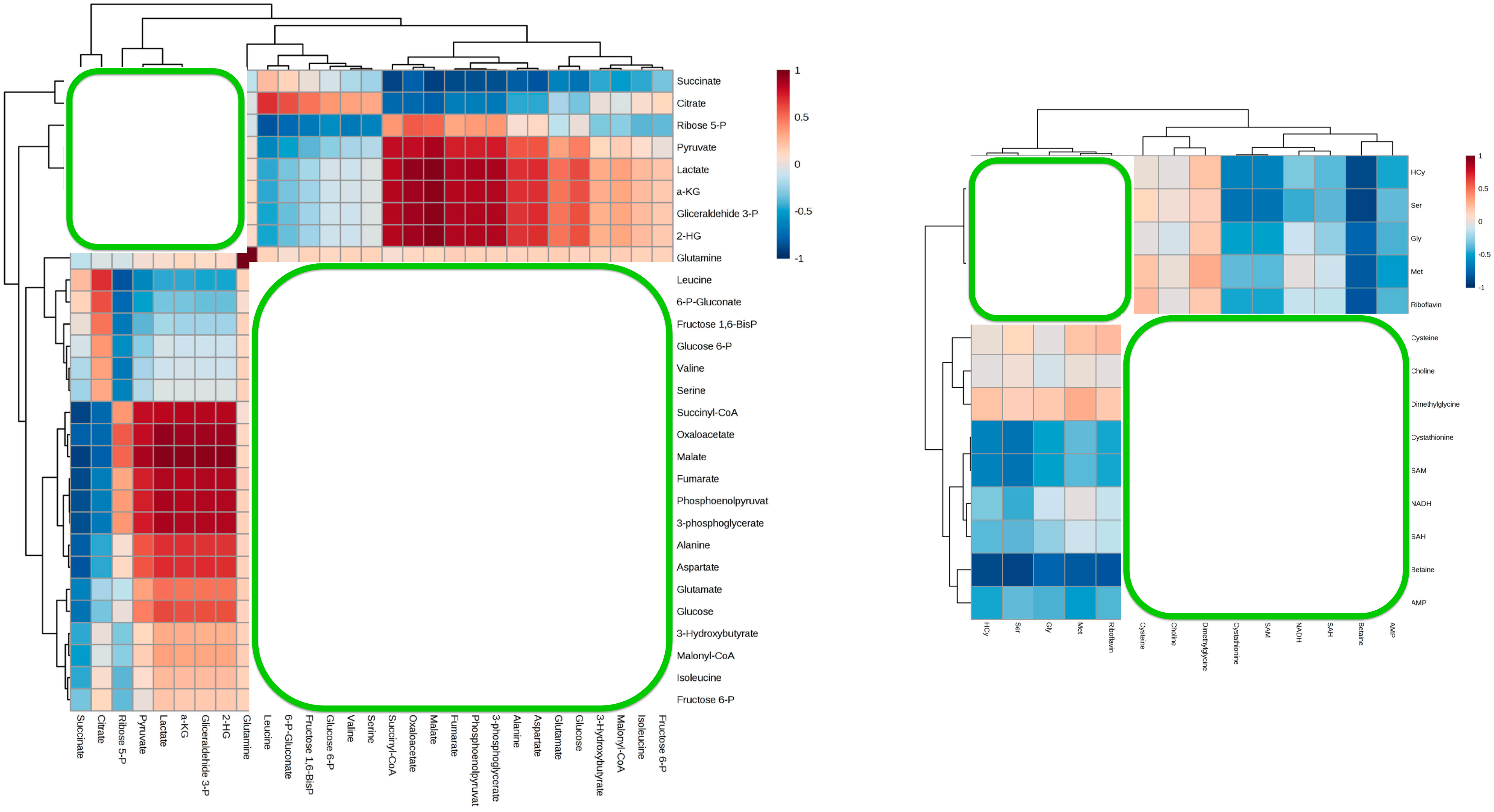

Figure S1. Correlation of the metabolomic data. Correlation matrix between all bioenergetic (*left*) and homocysteine-methionine 1C (*right*) metabolites collected from HMLE/HMLE-EMT and HMLER/HMLER-EMT cells based on the game's metrics. The color correlates to the intensity of metabolite concentration, where red indicates high intensity and blue indicates low intensity.
